# Urinary angiotensinogen level is associated with potassium homeostasis and clinical outcome in patients with polycystic kidney disease: a prospective cohort study

**DOI:** 10.1186/s12882-019-1292-3

**Published:** 2019-03-25

**Authors:** Hyoungnae Kim, Seohyun Park, Jong Hyun Jhee, Hae-Ryong Yun, Jung Tak Park, Seung Hyeok Han, Joongyub Lee, Soo Wan Kim, Yeong Hoon Kim, Yun Kyu Oh, Shin-Wook Kang, Kyu Hun Choi, Tae-Hyun Yoo, Representing the KNOW-CKD Investigators Group

**Affiliations:** 1 Department of Internal Medicine, College of Medicine, Institute of Kidney Disease Research, Yonsei University, Seoul, 03722 Republic of Korea; 2 Division of Nephrology, Soonchunhyang University Hospital, Seoul, Republic of Korea; 3 Division of Nephrology and Hypertension, Department of Internal Medicine, Inha University College of Medicine, Incheon, Republic of Korea; 4 Medical Research Collaborating Center, Seoul National University Hospital and Seoul National University College of Medicine, Seoul, Republic of Korea; 5 Department of Internal Medicine, Chonnam National University Medical School, Gwangju, Republic of Korea; 6 Department of Internal Medicine, Busan Paik Hospital, Inje University, Busan, Republic of Korea; 7 Department of Internal Medicine, Seoul National University College of Medicine, Boramae Medical Center, Seoul, Republic of Korea

**Keywords:** Polycystic kidney disease, Angiotensinogen, Potassium

## Abstract

**Background:**

Guidelines for general hypertension treatment do not recommend the combined use of renin-angiotensin-aldosterone system (RAAS) inhibitors due to the risk of hyperkalemia. However, a recent clinical trial showed that polycystic kidney disease (PKD) patients had infrequent episodes of hyperkalemia despite receiving combined RAAS inhibitors. Because intrarenal RAAS is a main component for renal potassium handling, we further investigated the association between intrarenal RAAS activity and serum potassium level in patients with chronic kidney disease, particularly in PKD patients, and examined whether intrarenal RAAS activity has a prognostic role in patients with PKD.

**Methods:**

A total of 1788 subjects from the KoreaN cohort study for Outcome in patients With Chronic Kidney Disease (KNOW-CKD) were enrolled in this study. Intrarenal RAAS activity was assessed by the measurement of urinary angiotensinogen (AGT). The primary outcome was the composite of all-cause mortality and renal function decline.

**Results:**

Patients with PKD had a significantly lower serum potassium level in chronic kidney disease stages 1 to 3b than non-PKD patients. In logistic regression analysis, after adjusting for multiple confounders, PKD patients had a significantly lower risk of hyperkalemia than non-PKD patients. In multivariable linear regression analysis, the urinary AGT/creatinine (Cr) ratio was negatively correlated with the serum potassium level (*β* = − 0.058, *P* = 0.017) and positively correlated with the transtubular potassium gradient (TTKG, *β* = 0.087, *P* = 0.001). In propensity score matching analysis, after matching factors associated with serum potassium and TTKG, PKD patients had a significantly higher TTKG (*P* = 0.021) despite a lower serum potassium level (*P* = 0.004). Additionally, the urinary AGT/Cr ratio was significantly higher in PKD patients than in non-PKD patients (*P* = 0.011). In 293 patients with PKD, high urinary AGT/Cr ratio was associated with increased risk of the composite outcome (hazard ratio 1.29; 95% confidence interval, 1.07–1.55; *P* = 0.007).

**Conclusions:**

High activity of intrarenal RAAS is associated with increased urinary potassium excretion and low serum potassium level in patients with PKD. In addition, intrarenal RAAS activity can be a prognostic marker for mortality and renal function decline in these patients.

## Background

Polycystic kidney disease (PKD) is a genetic disorder characterized by a progressively increasing number of fluid-filled cysts, distortion of the normal kidney structure, and loss of renal function over a period of decades [1]. Early-onset hypertension is one of the key features in PKD, and up to 80% of patients are diagnosed as having hypertension before significant renal dysfunction [2]. In addition, hypertension is associated with a larger kidney volume, progression to end-stage renal disease, and cardiovascular mortality in patients with PKD [3–5]. Although the pathophysiologic mechanism of hypertension has not been fully elucidated in PKD, it is generally accepted that the renin-angiotensin-aldosterone system (RAAS) plays a pivotal role [6, 7]. Moreover, RAAS is known to be one of the main contributors to the progression of chronic kidney disease (CKD). Numerous clinical trials have demonstrated that RAAS inhibition by an angiotensin-converting enzyme inhibitor (ACEi) or angiotensin-II receptor blocker (ARB) prevents the progression of CKD [8–11]. Thus, it was expected that dual inhibition using an ACEi and ARB would be more effective in inhibiting the progression of CKD; however, three randomized controlled trials have failed to demonstrate this [12–14]. In contrast, these trials demonstrated that dual inhibition significantly increased the incidence of hyperkalemia and renal impairment. Furthermore, two of the trials involving dual RAAS inhibition in patients with type 2 diabetes mellitus (DM) were terminated early because of an increased risk of hyperkalemia and acute kidney injury [13, 14].

Recently, the effect of dual RAAS inhibition in patients with PKD using an ACEi and ARB was evaluated in large randomized controlled trials (the HALT Progression of Polycystic Kidney Disease (HALT-PKD) studies) [15, 16]. These studies confirmed that reducing blood pressure is effective in reducing proteinuria and slowing the rate of total kidney volume increase. Interestingly, adverse events including hyperkalemia and acute kidney injury were infrequent and not different between the dual and single RAAS inhibition groups.

The potassium concentration in extracellular fluid is regulated within a narrow range by the kidney, the primary organ of this homeostatic system. Augmented activity of the RAAS increases sodium reabsorption in the kidney, which induces the electrochemical gradient for potassium to pass into the lumen in the distal convoluted tubule and collecting duct, enhancing potassium excretion [17]. Therefore in this study, we aimed to evaluate potassium regulation in patients with CKD due to PKD and other etiologies by analyzing the activity of the intrarenal RAAS and consequent tubular potassium secretion. Since urinary angiotensinogen (AGT) has been used as a valuable biomarker for estimating the intrarenal RAAS in patients with CKD [18], we compared the relationship between urinary AGT and serum potassium levels among patients with various etiologies of CKD. Furthermore, we investigated the role of urinary AGT as a prognostic marker in patients with PKD.

## Methods

### Study design and population

**Fig. 1:**
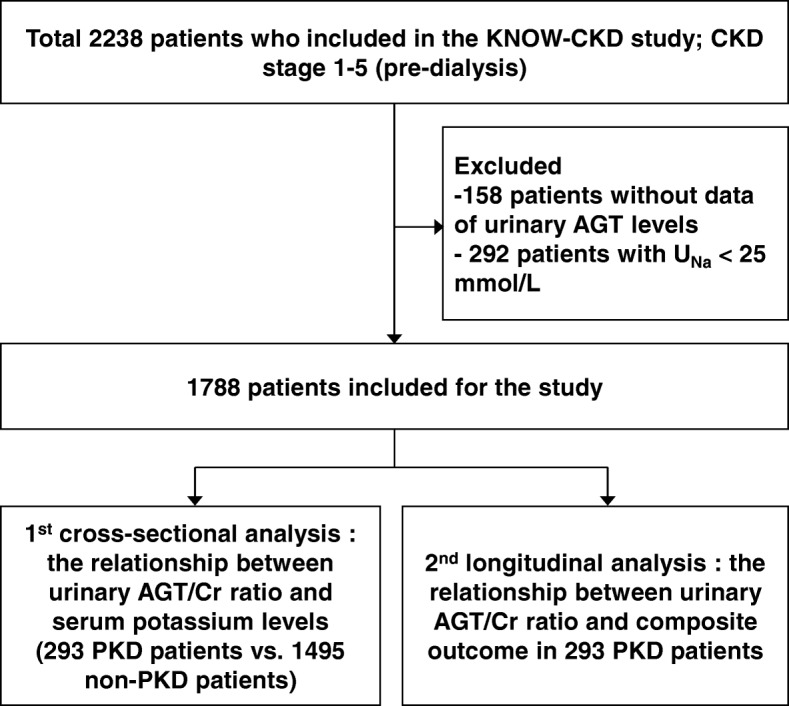
Flow chart for patient enrollment and analyses

We utilized data from the KoreaN cohort study for Outcome in patients With Chronic Kidney Disease (KNOW-CKD), a nationwide, multi-center, prospective cohort study to clarify the natural course, complication profiles, and risk factors of Asian populations with CKD. The detailed design and methods of the study have been described previously [19] (ID no. NCT01630486, http://www.clinicaltrials.gov). Briefly, 2341 individuals aged 20 to 75 years with CKD stages 1 to 5 without dialysis who voluntarily provided informed consent were recruited between 2011 and 2015 from 9 clinical centers. Exclusion criteria were as follows: 1) individuals unable or unwilling to give written consent, 2) individuals who previously received chronic dialysis or organ transplantation, 3) individuals with heart failure (New York Heart Association class III or IV), 4) individuals with liver cirrhosis (Child-Pugh class 2 or 3), 4) individuals with a past or current history of malignancy, 5) pregnant individuals, and 6) individuals with a single kidney due to trauma or kidney donation. After excluding 103 patients who did not meet the inclusion criteria or had missing data for isotope dilution mass spectrometry (IDMS)-calibrated creatinine (Cr), 2238 participants were included. For this study, we additionally excluded 158 patients without data for urinary AGT levels. Furthermore, to ensure correct calculation of the transtubular potassium gradient (TTKG), we excluded another 292 patients with random urine sodium less than 25 mmol/L and those with urine osmolality less than the plasma osmolality [20]. Finally, 1788 patients were included in the analysis. We first analyzed the relationship between urinary AGT and serum potassium level according to etiology of CKD in 1788 patients (Fig. 1). In addition, we conducted a longitudinal analysis in 293 PKD patients to evaluate the impact of urinary AGT level on outcome in this group. This study was carried out in accordance with the Declaration of Helsinki, and the study protocol was approved by the respective institutional review boards of the participating centers, including Seoul National University Hospital, Yonsei University Severance Hospital, Kangbuk Samsung Medical Center, Seoul St. Mary’s Hospital, Gil Hospital, Eulji General Hospital, Chonnam National University Hospital, and Busan Paik Hospital.

### Data collection

Baseline sociodemographic data were retrieved from the electronic data management system of the KNOW-CKD developed by the Seoul National University Medical Research Collaborating Center. Anthropometric measurements, including height and weight, were obtained at the baseline visit, and blood pressure was measured using an electronic sphygmomanometer by a trained nurse in the clinic. Blood and first-voided urine samples were sent to the central laboratory of the KNOW-CKD (Lab Genomics, Seongnam, Republic of Korea) by standard protocol. The serum Cr level was measured by using the IDMS-traceable method, and estimated glomerular filtration rate (eGFR) was calculated with the CKD Epidemiology Collaboration equation [21]. The urinary AGT level was measured by using an enzyme-linked immunosorbent assay kit (IBL International GmBH, Hamburg, Germany). Intra-assay and inter-assay coefficients of variation were less than 5.5 and 5.8%, respectively. The TTKG was calculated by using the following formula: (urine potassium level/serum potassium level)/(urine osmolality/serum osmolality) [22]. Hyperkalemia was defined as a serum potassium level greater than 5.0 mmol/L.

### Clinical outcome

The primary outcome of this study was the composite of all-cause mortality and renal function decline. Renal function decline was defined as a > 50% decline of the eGFR from baseline, doubling of the serum creatinine level, or the initiation of dialysis (hemodialysis or peritoneal dialysis).

### Statistical analyses

All subjects were categorized into 5 groups according to the etiology of CKD: diabetic nephropathy (DN), hypertensive nephrosclerosis (HTN), glomerulonephritis (GN), polycystic kidney disease (PKD), and unclassified. Continuous variables between groups were compared using one-way analysis of variance, and post-hoc analyses between groups were conducted with Bonferroni analysis. Nonparametric variables were compared using the Kruskal-Wallis test, and categorical variables were compared using the chi-square test. The risks of hyperkalemia among CKD patients grouped by etiology were compared using multivariable logistic regression analysis. To evaluate the relationship between the urinary AGT/Cr ratio and serum potassium level or TTKG, multivariable linear regression analyses were conducted, and the selection of covariables was done using the stepwise method. In addition, to minimize the effects of comorbidities, kidney function, and other laboratory findings, propensity score matching (PSM) was used. Propensity scores were estimated using logistic regression with the nearest neighbor technique without replacement, and a predefined caliper of 0.2 times the standard deviation. The covariables shown to be associated with the serum potassium level and TTKG in multivariable linear regression analysis were used for the matching. The PKD group was matched with the non-PKD group at a ratio of 1:1. In these matched cohorts, comparisons between groups were conducted with the paired t-test, McNemar’s test, and Wilcoxon signed-rank test, as appropriate. Finally, we conducted multivariable Cox regression analysis to determine whether the AGT/Cr ratio was a prognostic factor of composite outcome in all 293 patients with PKD. We further categorized patients into 2 groups according to the median value of AGT/Cr ratio, and delineated the cumulative hazard of composite outcome in them using the multivariable Cox regression model. Statistical significance was defined as *P* < 0.05. All statistical analyses were conducted by using SPSS software, version 23.0 with Essentials for R Plug-in (IBM Corporation, Armonk, NY, USA).

## Results

### Baseline characteristics

The baseline characteristics of patients according to the etiology of CKD are presented in Table 1. Patients’ mean age was 54 ± 12.2 years, and 38.6% were women. The mean age was significantly lower in the PKD group than in the other groups. The prevalence rates of hypertension and DM were significantly lower in the PKD group than in the other groups (*P* < 0.001, all). The mean creatinine level and eGFR were 1.8 mg/dL and 50.1 mL/min/1.73 m^2^, respectively. The mean eGFR was significantly higher in the PKD group than in the other groups (*P* < 0.001, all). The median urinary AGT/Cr ratio was 32.5, and it was not significantly different between the groups. However, the mean serum potassium level in the PKD group was 4.4 ± 0.4 mmol/L, which was lower than that of the other groups (*P* < 0.001, all). In addition, the mean TTKG was significantly higher in the PKD group than in the other groups (*P* < 0.001, all). Fewer patients in the PKD group received any RAAS inhibition than the DN (*P* = 0.001) and GN (*P* < 0.001) groups, and fewer patients in the PKD group received dual RAAS inhibition than the others as well. The PKD group also had a smaller number of patients who received diuretics compared to the other groups (*P* < 0.001, all).

**Table 1:** Baseline characteristics of patients according to etiology of CKD

5.Variables	Total	Subcohort					*p*-value
		PKD	DN	HTN	GN	Unclassified	
Participants	1788	293	429	334	618	114	
Age (years)	54.0 ± 12.2	47.0 ± 10.9	59.5 ± 9.2^‡^	59.8 ± 10.9^‡^	50.1 ± 12.1^‡^	54.9 ± 13.1^‡^	< 0.001
Female (n,%)	690 (38.6)	148 (50.5)	127 (29.6) ^‡^	93 (27.8) ^‡^	273 (44.2)	49 (43.0)	< 0.001
Hypertension (n,%)	1723 (96.4)	255 (87.0)	424 (98.8) ^‡^	334 (100.0) ^‡^	600 (97.1) ^‡^	110 (96.5) ^‡^	< 0.001
DM (*n,*%)	628 (35.1)	12 (4.1)	429 (100.0) ^‡^	63 (18.9) ^‡^	57 (9.2) ^‡^	67 (58.8) ^‡^	< 0.001
Current smoker (*n*,%)	272 (15.2)	40 (13.7)	70 (16.3) ^‡^	63 (18.9) ^‡^	80 (12.9)	19 (16.7)	< 0.001
BMI (kg/m^2^)	24.7 ± 3.4	23.6 ± 3.1	25.4 ± 3.3^‡^	25.3 ± 3.5^‡^	24.2 ± 3.3	25.5 ± 4.0^‡^	< 0.001
Cardiovascular disease (*n*, %)							
MI	33 (1.8)	1 (0.3)	18 (4.2) ^‡^	8 (2.4) ^‡^	\5 (0.8)	1 (0.9)	< 0.001
Stroke	115 (6.4)	17 (5.8)	44 (10.3) ^‡^	34 (10.2) ^‡^	15 (2.4) ^‡^	5 (4.4)	< 0.001
PAD	68 (3.8)	0 (0.0)	29 (6.8) ^‡^	17 (5.1) ^‡^	10 (1.6) ^‡^	12 (10.5) ^‡^	< 0.001
SBP (mmHg)	128.9 ± 16.3	128.7 ± 13.3	135.4 ± 18.1^‡^	128.9 ± 15.4	124.1 ± 14.6^‡^	131.0 ± 19.5	< 0.001
DBP (mmHg)	76.9 ± 11.1	80.5 ± 10.2	75.9 ± 11.6^‡^	77.8 ± 11.4^‡^	75.4 ± 10.0^‡^	77.5 ± 13.4	< 0.001
CKD stages (*n*, %)							
Stage 1	204 (11.4)	76 (25.9)	12 (2.8)	9 (2.7)	91 (14.7)	16 (14.0)	< 0.001
Stage 2	326 (18.2)	90 (30.7)	30 (7.0)	37 (11.1)	141 (22.8)	28 (24.6)	
Stage 3a	326 (18.2)	43 (14.7)	65 (15.2)	77 (23.1)	127 (20.6)	14 (12.3)	
Stage 3b	400 (22.4)	39 (13.3)	110 (25.6)	94 (28.1)	128 (20.7)	29 (25.4)	
Stage 4	424 (23.7)	34 (11.6)	167 (38.9)	97 (29.0)	105 (17.0)	21 (18.4)	
Stage 5	108 (6.0)	11 (3.8)	45 (10.5)	20 (6.0)	26 (4.2)	6 (5.3)	
Creatinine (mg/dL)	1.8 ± 1.1	1.3 ± 0.9	2.3 ± 1.3^‡^	2.0 ± 1.2^‡^	1.6 ± 1.0^‡^	1.6 ± 0.9	< 0.001
eGFR (mL/min/1. 73m^2^)	50.1 ± 29.9	68.1 ± 34.3	35.3 ± 20.4^‡^	40.3 ± 21.0^‡^	56.2 ± 30.3^‡^	54.6 ± 31.9^‡^	< 0.001
Hemoglobin (g/dL)	12.8 ± 2.0	13.3 ± 1.8	11.7 ± 1.9^‡^	13.3 ± 2.0	13.2 ± 1.9	12.8 ± 2.3	< 0.001
Albumin (g/dL)	4.2 ± 0.4	4.4 ± 0.3	4.0 ± 0.5^‡^	4.3 ± 0.3	4.1 ± 0.4^‡^	4.2 ± 0.5^‡^	< 0.001
Calcium (mg/dL)	9.1 ± 0.5	9.3 ± 0.5	8.9 ± 0.6^‡^	9.2 ± 0.5	9.2 ± 0.5^‡^	9.2 ± 0.5	< 0.001
Phosphorus (mg/dL)	3.7 ± 0.7	3.6 ± 0.6	3.9 ± 0.7^‡^	3.6 ± 0.6	3.6 ± 0.6	3.8 ± 0.6	< 0.001
Sodium (mmol/L)	141.0 ± 2.3	141.1 ± 2.2	140.8 ± 2.7	141.1 ± 2.3	141.0 ± 2.2	140.4 ± 2.4	0.037
Potassium (mmol/L)	4.6 ± 0.6	4.4 ± 0.4	4.9 ± 0.6^‡^	4.6 ± 0.6^‡^	4.6 ± 0.5^‡^	4.7 ± 0.6^‡^	< 0.001
Chloride (mmol/L)	105.6 ± 3.5	105.6 ± 2.9	106.1 ± 4.2	105.6 ± 3.5	105.4 ± 3.3	105.1 ± 3.7	0.011
Total CO_2_ (mmol/L)	25.7 ± 3.6	26.6 ± 3.2	24.9 ± 3.5^‡^	25.5 ± 3.6^‡^	26.1 ± 3.7	25.5 ± 3.8	< 0.001
LDL cholesterol (mg/dL)	96.5 ± 31.0	101.7 ± 27.6	89.8 ± 32.6^‡^	93.8 ± 30.1^‡^	100.1 ± 30.7	96.7 ± 32.0	< 0.001
HDL cholesterol (mg/dL)	49.1 ± 15.2	54.6 ± 14.0	43.1 ± 13.2^‡^	46.3 ± 13.9^‡^	51.6 ± 15.6^‡^	52.0 ± 17.5	< 0.001
UPCR (g/g)*	0.5 (0.1–1.5)	0.8 (0.5–2.0)	1.6 (0.4–3.8) ^‡^	0.3 (0.1–0.8) ^‡^	0.6 (0.3–1.6) ^‡^	0.6 (0.2–1.6) ^‡^	< 0.001
UACR (mg/g)*	347.9 (77.9-1080.5)	35.2 (13.3–121.7)	1153.3^‡^ (290.8-2642.9)	175.2^‡^ (24.9–535.1)	483.7^‡^ (216.2-1157.7)	404.6^‡^ (102.0-1127.3)	< 0.001
Urine AGT/Cr ratio (μg/g)*	32.5 (9.2-139.3)	30.5 (12.5–82.7)	35.1 (6.8–232.7)	29.3 (9.9–123.4)	36.0 (9.0–137.9)	37.9 (8.4–205.3)	0.719
Urine osmolality (mOsm/kg)	511.4 ± 144.8	516.7 ± 156.2	462.9 ± 108.3^‡^	504.8 ± 129.7	545.9 ± 159.3^‡^	513.3 ± 142.6	< 0.001
TTKG	6.4 ± 2.4	7.2 ± 2.8	5.6 ± 2.1^‡^	6.3 ± 2.3^‡^	6.5 ± 2.4^‡^	6.3 ± 2.4^‡^	< 0.001
RAAS blockade (n, %)							
Total	1539 (86.1)	229 (78.2)	375 (87.4) ^‡^	278 (83.2)	559 (90.6) ^‡^	98 (86.0)	< 0.001
ARB only	1344 (75.2)	215 (73.4)	325 (75.8)	254 (76.0)	462 (74.9)	88 (77.2)	0.91
ACEi only	96 (5.4)	12 (4.1)	24 (5.6)	11 (3.3)	43 (7.0)	6 (5.3)	0.14
Dual blockade	99 (5.5)	2 (0.7)	26 (6.1) ^‡^	13 (3.9) ^‡^	54 (8.8) ^‡^	4 (3.5) ^‡^	< 0.001
RAAS blockade dose (mg)							
ARB^†^	68.5 ± 32.2	68.9 ± 25.2	73.7 ± 34.3	66.6 ± 32.2	66.1 ± 31.2	67.0 ± 36.0	0.01
ACEi^¶^	8.9 ± 5.3	8.9 ± 5.3	10.6 ± 6.1	14.1 ± 10.4	10.2 ± 5.1	8.9 ± 5.3	0.051
Diuretics (n,%)	577 (32.3)	32 (10.9)	245 (57.1) ^‡^	115 (34.4) ^‡^	151 (24.5) ^‡^	34 (29.8) ^‡^	< 0.001
Diuretics dose (mg)							
Furosemide	39.1 ± 25.0	26.7 ± 10.3	42.0 ± 28.6	32.8 ± 15.3	38.0 ± 22.4	37.1 ± 15.4	0.145
Torsemide	6.5 ± 7.8	-	4.8 ± 2.2	6.7 ± 2.6	4.8 ± 1.8	14.5 ± 20.0	0.161
Hydrochlorothiazide	14.2 ± 4.9	14.8 ± 5.5	14.7 ± 6.3	14.2 ± 4.3	13.9 ± 3.9	11.9 ± 2.0	0.469
Spironolactone	27.2 ± 11.0	25.0 ± 0.0	37.5 ± 17.7	33.3 ± 14.4	25.0 ± 7.9	26.6 ± 18.0	0.53
Calcium channel blocker (n, %)	750 (42.0)	101 (34.5)	240 (55.9) ^‡^	169 (50.6) ^‡^	184 (29.8)	56 (49.1) ^‡^	< 0.001
Beta blocker (n, %)	457 (25.6)	72 (24.6)	155 (36.1) ^‡^	108 (32.3) ^‡^	92 (14.9) ^‡^	30 (26.3)	< 0.001

*Data are expressed as median and interquartile ranges

^‡^Statistically different (P < 0.05) when compared to PKD group

^†^All other doses of ARBs were substituted for the equivalent dose of losartan

^¶^All other doses of ACE inhibitors were substituted for the equivalent dose of enalapril

*Abbreviations*: CKD, chronic kidney disease; DN, diabetic nephropathy; HTN, hypertensive nephropathy; GN, glomerulonephritis; PKD, polycystic kidney disease; DM, diabetes mellitus; BMI, body mass index; CAD, coronary arterial disease; MI, myocardial infarction; PAD, peripheral arterial disease; SBP, systolic blood pressure; DBP, diastolic blood pressure; eGFR, estimated glomerular filtration rate; LDL, low-density lipoprotein; HDL, high-density lipoprotein; AGT, angiotensinogen; TTKG, transtubular potassium gradient; RAAS, renin-angiotensin-aldoterone system; ARB, angiotensin receptor blocker; ACEi, angiotensin converting enzyme inhibitor

### Risk of hyperkalemia among CKD groups

**Fig. 2:**
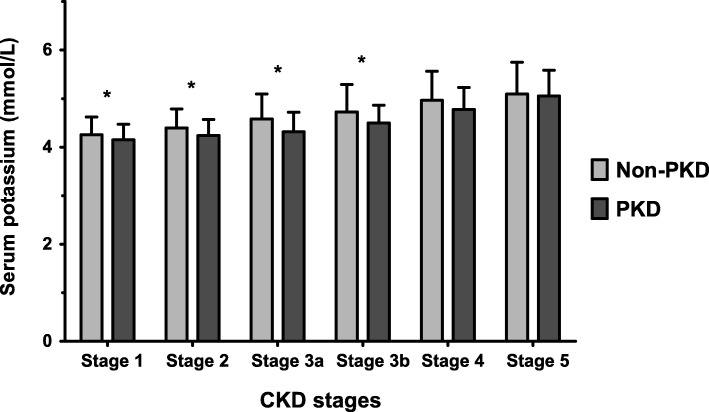
Serum potassium levels in each CKD stage. * *P* < 0.05

**Fig. 3:**
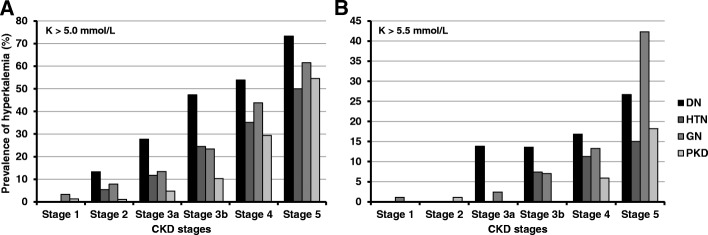
Prevalence of hyperkalemia according to etiology of CKD in each CKD stage. **a** serum potassium level > 5.0 mmol/L, (**b**) serum potassium level > 5.5 mmol/L.

When we compared serum potassium levels for each CKD stage, the PKD group had a lower serum potassium level in CKD stages 1 to 3 than the non-PKD group (Fig. 2). The prevalence of hyperkalemia was also lower in the PKD group than in the other CKD groups up to CKD stage 3 (K > 5.0) and stage 4 (K > 5.5). In the other stages it is similar to all except DN (K > 5.0) or HTN (K > 5.5) (Fig. 3). The lower prevalence of hyperkalemia in PKD was prominent in the early CKD stages, and attenuated with decline of renal function. In the multivariable logistic regression analysis, we built up consecutive multi-step models. First, we adjusted for age, sex, history of DM, body mass index (BMI), and systolic blood pressure (SBP) in model 1, and found that the risk of hyperkalemia was significantly lower in the PKD group than in the other CKD groups (Table 2). In model 2, we further adjusted for laboratory parameters including serum sodium and urinary protein-to-creatinine ratio (UPCR); use of anti-hypertensive medications (RAAS inhibition, diuretic, calcium channel blocker, and beta blocker) was then additionally adjusted for in model 3. Significantly lower risk of hyperkalemia in PKD was persistent in all models.

**Table 2:** Logistic regression analysis for the risk of hyperkalemia according to etiology of CKD

CKD subcohort	Unadjusted		Model 1		Model 2		Model 3	
	OR (95% CI)	*p*-value	OR (95% CI)	*p*-value	OR (95% CI)	*p*-value	OR (95% CI)	*p*-value
PKD	(Reference)		(Reference)		(Reference)		(Reference)	
DN	9.52 (6.02–15.06)	< 0.001	8.77 (4.74–16.24)	< 0.001	4.65 (2.42–8.93)	< 0.001	4.91 (2.54–9.50)	< 0.001
HTN	3.42 (2.10–5.57)	< 0.001	3.47 (2.07–5.83)	< 0.001	2.54 (1.50–4.30)	0.001	2.57 (1.51–4.37)	< 0.001
GN	2.79 (1.76–4.42)	< 0.001	2.92 (1.82–4.66)	< 0.001	1.70 (1.03–2.81)	0.038	1.82 (1.10–3.03)	0.02
Unclassified	4.57 (2.55–8.17)	< 0.001	4.59 (2.44–8.65)	< 0.001	2.86 (1.48–5.53)	0.002	2.95 (1.52–5.72)	0.001

Model 1: adjusted age, sex, history of DM, BMI, and SBP

Model 2: Model 1 + serum sodium and UPCR*

Model 3: Model 2 + use of RAAS blockade, diuretics, CCB, and beta blockers

*Data were log transformed

*Abbreviations*: *CKD* Chronic kidney disease, *OR* odds ratio, *CI* Confidence interval, *PKD* Polycystic kidney disease, *DN* Diabetic nephropathy, *HTN* Hypertensive nephrosclerosis, *GN*, glomerulonephritis; eGFR, estimated glomerular filtration rate; *BMI* Body mass index; SBP, systolic blood pressure; UPCR, urine protein-to-creatinine ratio; RAAS, renin-angiotensin-aldosterone system; CCB, calcium channel blocker

### Urinary AGT/Cr ratio was correlated with the serum potassium level and TTKG

To evaluate the association between intrarenal RAAS activity and the serum potassium level, multivariable linear regression analyses were conducted (Table 3). In these analyses, a history of DM, BMI, eGFR, total carbon dioxide (CO_2_) level, serum albumin level, UPCR, urine osmolality, and use of diuretics were significantly correlated with the serum potassium level. In addition, the eGFR, total CO_2_ level, urine osmolality, age, sex, and Charlson comorbidity index (CCI) were associated with TTKG. The urinary AGT/Cr ratio was negatively correlated with the serum potassium level (*β* = − 0.058, *P* = 0.017) and positively correlated with TTKG (*β* = 0.087, *P* = 0.001).

**Table 3:** Multivariate linear regression analyses for the relationship between serum potassium, transtubular potassium gradient, and urine angiotensinogen-creatinine ratio

Variables	Dependent variables			
	Serum potassium		TTKG	
	*β*	*p*-value	*β*	*p*-value
DM (vs. non-DM)	0.171	< 0.001	–	–
BMI (kg/m^2^)	−0.047	0.044	–	–
eGFR (mL/min/1. 73m^2^)	−0.315	< 0.001	0.157	< 0.001
Total CO_2_ (mmol/L)	−0.152	< 0.001	0.174	< 0.001
Serum albumin (g/dL)	0.066	0.019	–	–
UPCR (g/g)*	0.120	< 0.001	–	–
Urine AGT/Cr ratio (μg/g)*	− 0.058	0.017	0.087	0.001
Urine osmolality (mOsm/kg)*	− 0.056	0.046	0.118	< 0.001
Diuretics (vs. non-user)			–	–
Age (years)	–	–	0.104	< 0.001
Sex (vs. female)	–	–	0.139	< 0.001
Charlson comorbidity index	–	–	−1.000	0.002

*β*: Standardized coefficient

*Variables are log transformed

*Abbreviations*: TTKG, trans-tubular potassium gradient; DM, diabetes mellitus; BMI, body mass index; eGFR, estimated glomerular filtration; CO2, carbon dioxide; UPCR, urine protein-to-creatinine ratio; AGT/Cr, angiotensinogen/creatinine

### Values of urinary AGT/Cr ratio, serum potassium, and TTKG after PSM

To minimize the effect of confounders, PSM was performed. The matching was conducted with covariables, including age, sex, CCI, DM, BMI, eGFR, total CO_2_ level, serum albumin level, UPCR, urine osmolality, and use of diuretics, which were shown to be associated with the serum potassium level and TTKG. After matching patients in the PKD group with those in the non-PKD group, 196 patients remained in each group (Table 4). In this matched cohort, patients in the PKD group had a significantly lower serum potassium level (*P* = 0.004) than the non-PKD group, whereas the serum sodium (*P* = 0.007) and chloride (*P* = 0.002) levels were higher in the PKD group than in the non-PKD group. Interestingly, the TTKG was significantly higher in the PKD group (*P* = 0.021), which means that urinary excretion of potassium was larger despite lower serum potassium levels in the PKD group. Moreover, the percentage of patients who received RAAS inhibition was not different between groups in the matched cohort, however, patients in the PKD group had a significantly higher urinary AGT/Cr ratio (*P* = 0.011).

**Table 4:** Propensity score matching analysis between the PKD and the non-PKD

Variables	Before PSM			After PSM		
	Non-PKD (*N* = 1495)	PKD (*N* = 293)	*p*-value	Non-PKD (*N* = 196)	PKD (*N* = 196)	*p*-value
Matched variables						
Age (years)	55.3 ± 12.0	47.0 ± 10.9	< 0.001	49.6 ± 12.9	49.1 ± 11.1	0.66
Female (n, %)	542 (36.3)	148 (50.5)	< 0.001	87 (44.4)	87 (44.4)	> 0.999
Charlson comorbidity index	1.6 ± 1.3	1.4 ± 1.2	0.125	1.6 ± 1.3	1.4 ± 1.2	0.098
DM (n, %)	616 (41.2)	12 (4.1)	< 0.001	22 (11.2)	11 (5.6)	0.068
BMI (kg/m^2^)	24.9 ± 3.4	23.6 ± 3.1	< 0.001	24.2 ± 3.7	24.1 ± 3.2	0.891
eGFR (mL/min/1. 73m^2^)	46.6 ± 27.6	68.1 ± 34.2	< 0.001	57.7 ± 29.7	59.1 ± 30.1	0.589
Total CO_2_ (mmol/L)	25.6 ± 3.7	26.6 ± 3.2	< 0.001	26.4 ± 3.2	26.3 ± 3.2	0.924
Serum albumin (g/dL)	4.1 ± 0.4	4.4 ± 0.3	< 0.001	4.3 ± 0.3	4.4 ± 0.3	0.39
UPCR (g/g)^a^	0.6 (0.2–1.9)	0.1 (0.0–0.2)	< 0.001	0.1 (0.0–0.4)	0.1 (0.0–0.3)	0.099
Urine osmolality (mOsm/kg)^a^	510.4 ± 142.5	516.7 ± 156.2	0.499	513.3 ± 148.0	514.2 ± 160.8	0.845
Diuretics (n, %)	545 (36.5)	32 (10.9)	< 0.001	29 (14.8)	26 (13.3)	0.663
Matching results						
Sodium (mmol/L)	140.9 ± 2.4	141.1 ± 2.2	0.206	140.9 ± 2.2	141.5 ± 2.0	0.007
Chloride (mmol/L)	105.6 ± 3.7	105.6 ± 3.0	0.716	105.1 ± 3.4	106.1 ± 3.0	0.002
Potassium (mmol/L)	4.7 ± 0.6	4.4 ± 0.4	< 0.001	4.6 ± 0.5	4.4 ± 0.5	0.004
TTKG	6.2 ± 2.3	7.2 ± 2.8	< 0.001	6.5 ± 2.6	7.1 ± 2.4	0.021
Urine AGT/Cr ratio (μg/g)^a^	33.6 (8.6–157.4)	30.5 (12.5–82.7)	0.627	17.7 (8.2–63.1)	30.5 (13.0–109.5)	0.011
RAAS blockade (n, %)	1310 (87.7)	229 (78.2)	< 0.001	167 (85.2)	162 (82.7)	0.492

^a^Data are expressed as median and interquartile range. Comparison was done by Mann-Whitney U test before matching, and Wilcoxon signed-rank test after matching

*Abbreviations*: PKD, polycystic kidney disease; PSM, propensity score matching; DM, diabetes mellitus; BMI, body mass index; eGFR, estimated glomerular filtration rate; CO_2_, carbon dioxide; UPCR, urine protein-to-creatinine ratio; TTKG, transtubular potassium gradient; AGT/Cr, angiotensinogen/creatinine

### Urinary AGT/Cr ratio as a prognostic marker in patients with PKD

**Fig. 4:**
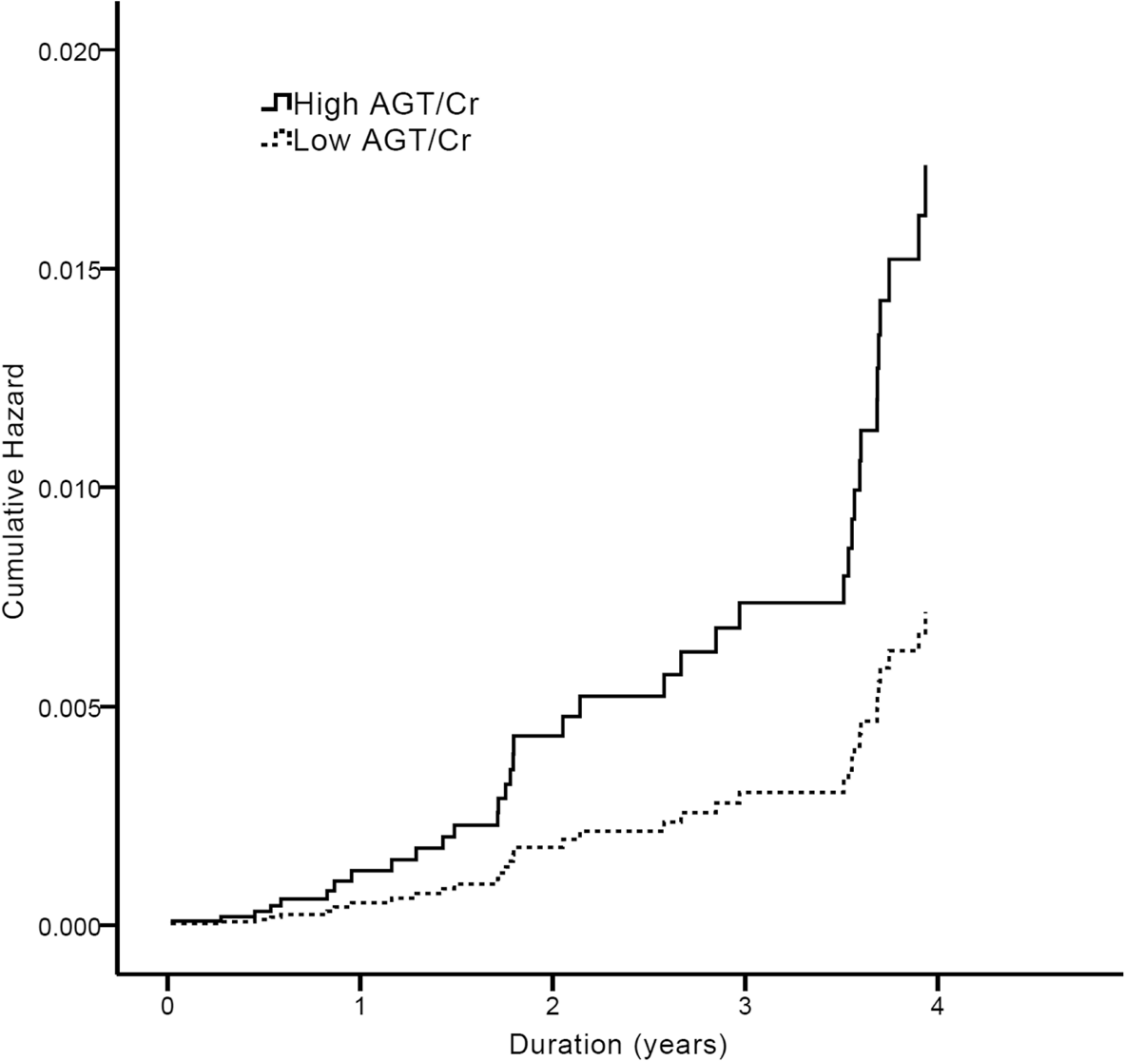
Cox proportional hazards regression curves for composite outcome in two groups of urinary AGT/Cr ratio. Curves were derived by adjustment of following covariables: age, sex, baseline eGFR, BMI, SBP (≥ 130 mmHg or not), presence of macro-albuminuria, use of RAAS blocker

To examine the role of the urinary AGT/Cr ratio as a prognostic factor in the PKD group, we conducted multivariable Cox regression analyses (Table 5). During the median follow-up of 4.6 years, 37 (12.6%) composite events (all-cause mortality and renal function decline) occurred. After adjusting for multiple covariables, the urinary AGT/Cr ratio was a significant risk factor for the composite outcome (hazard ratio (HR) 1.29; 95% confidence interval (CI), 1.07–1.55; *P* = 0.007). When we stratified patients into two groups according to the median value of the urinary AGT/Cr ratio of 30.5, those in the high urinary AGT/Cr ratio group had a significantly higher cumulative hazard of the composite outcome (HR 2.26; 95% CI, 1.10–4.65; *P* = 0.026; Fig. 4).

**Table 5:** Multivariable Cox regression analysis for composite outcome in patients with PKD

Variables	Hazard ratio	95% confidence interval	*p*-value
Age (year)	1.00	0.96–1.04	0.964
Sex (vs. female)	0.72	0.34–1.51	0.383
Baseline eGFR (mL/min/1. 73m^2^)	0.91	0.88–0.93	< 0.001
SBP (vs. < 130 mmHg)	2.02	1.03–3.97	0.042
BMI (kg/m^2^)	0.96	0.86–1.06	0.387
Use of RAAS blocker (vs. non-user)	1.29	0.42–3.95	0.655
Macroalbuminuria (vs. normo- or microalbuminuria)	1.40	0.56–3.48	0.468
Urine AGT/Cr ratio (μg/g)^a^	1.29	1.07–1.55	0.007

^a^Variable was log transformed

*Abbreviations*: eGFR, estimated glomerular filtration rate; SBP, systolic blood pressure; BMI, body mass index; RAAS, renin-angiotensin-aldosterone; AGT/Cr, angiotensinogen/creatinine

## Discussion

In this study, we demonstrated that the risk of hyperkalemia was significantly lower in patients with PKD than in patients with other etiologies of CKD. In addition, the urinary AGT/Cr ratio was associated with TTKG as well as the serum potassium level. By using PSM analysis, we found that patients with PKD had a significantly lower serum potassium level and a higher urinary AGT/Cr ratio and TTKG when compared to patients without PKD. Therefore, it can be presumed that high activity of intrarenal RAAS causes larger urinary potassium excretion which leads to lower serum potassium levels in patients with PKD. Furthermore, the urinary AGT/Cr ratio was a significant risk factor of all-cause mortality and decline in renal function in patients with PKD.

It has been widely accepted that increased RAAS activity is a central physiologic mechanism for the development of hypertension in patients with PKD, and recent studies have focused further on its role in patients with PKD. Among several intrarenal RAAS components, the urinary AGT level has been shown to be correlated with the intrarenal activities of AGT and angiotensin II [23, 24]. In addition, the urinary AGT/Cr ratio was inversely correlated with the eGFR and positively correlated with the height-adjusted total kidney volume in patients with PKD [25]. Kocyigit et al. also found that the urinary AGT/Cr ratio was significantly associated with SBP [26], and reported that it was higher in hypertensive patients with PKD than healthy controls. A recent study by Salih et al. compared circulating and urinary RAAS components between patients with PKD and those with CKD but without PKD [27]; after adjusting for sex, eGFR, blood pressure, and RAAS inhibitor use between groups, the urinary AGT level and renin excretion were 5- to 6-fold higher in PKD than non-PKD patients, whereas circulating levels were not different. This was consistent with the findings in our study. There have been several possible explanations to explain why patients with PKD have increased urinary RAAS activity. One is renin synthesis by the cyst epithelium and dilated tubules [28]. Another is that other components of RAAS, including AGT, angiotensin converting enzyme (ACE), and angiotensin II can be produced within cysts and several parts of the tubules [29]. In addition, because renin and AGT are reabsorbed by a megalin-dependent pathway in the proximal tubule [30, 31], the functional defect in the proximal tubule in PKD can lead to increased concentration of tubular renin and angiotensinogen. Considering that ACE is abundant in the proximal tubular brush border, the highly concentrated tubular renin and angiotensinogen can be easily converted to angiotensin I and II [32]. Moreover, augmented intrarenal RAAS activities are associated with chronic inflammation and fibrotic change in the kidney, which can lead to progressive renal injury [33, 34]. Accordingly, previous studies showed that urinary AGT was associated with the development and progression of CKD [18, 35]. In our study, we also showed that urinary AGT/Cr ratio was correlated with decline in renal function and mortality in patients with PKD. To our knowledge, this is the first longitudinal study that has shown urinary AGT as a prognostic marker in patients with PKD.

Of note, we additionally found that the AGT/Cr ratio was negatively correlated with serum potassium level and positively correlated with TTKG. In our PSM analysis, the TTKG was paradoxically elevated in patients with PKD, even though it should be lowered when the serum potassium level is reduced. TTKG is positively associated with mineralocorticoid activity [36]. Lai et al. previously reported that the prevalence of primary aldosteronism was 33% in patients with PKD, which was greater than that in the general population [37]. Therefore, augmented intrarenal activities of angiotensin II and aldosterone in PKD may lead to a lower serum potassium level than that in other etiologies of CKD. In the ONTARGET study, even though the mean creatinine level of the participants was within normal range, the combined use of telmisartan and ramipril was associated with a higher incidence of hyperkalemia [38]. In addition, combination treatment of losartan and lisinopril for patients with DM in CKD stages 2 and 3 was stopped early in another study owing to safety concerns, including hyperkalemia [13]. The combination of a direct renin inhibitor with other RAAS inhibitors also significantly increased the risk of hyperkalemia [14]. As a result, recent guidelines for hypertension do not recommend combined use of RAAS inhibitors in general hypertensive patients [39–41]. Furthermore, some researchers have been concerned about an increased risk of cancer with combination RAAS inhibitor therapy [42]. However, when confined to PKD patients, even in the study conducted for advanced PKD patients with an eGFR less than 60 mL/min/1.73 m^2^, episodes of hyperkalemia were infrequent, and cancer risk was not increased [15]. Taking these results together, it can be suggested that patients with PKD may have a lower risk of hyperkalemia than those with other etiologies of CKD. Considering that the HALT-PKD investigators used a dose-adjustment protocol of RAAS inhibitors to achieve a specific blood pressure target, a higher degree of RAAS inhibition may be beneficial for attenuating the decline in renal function in PKD patients with a low risk of hyperkalemia.

Some limitations of this study should be discussed. First, we cannot confirm causality based on our cross-sectional analyses. However, with the vigorous adjustment of covariables and diverse analyses in a large cohort, we found that patients with PKD had a higher AGT/Cr ratio and a lower serum potassium level than those with other etiologies of CKD. Second, our data did not encompass any parameter of systemic RAAS activity. Systemic AGT is produced and released into circulation by the liver. Since the molecular weight of AGT is 52–64 kDa and it is negatively charged (similar to albumin), it cannot be filtered in the glomerulus of a healthy kidney [32]. However, in the case of proteinuric patients, systemic AGT can be filtered through the glomerular barrier; thus, the measured urinary excretion of AGT may partially reflect hepatic production and not purely intrarenally produced AGT. Moreover, many experimental and clinical studies have reported that the urinary AGT/Cr ratio was positively correlated with proteinuria [18, 23, 26, 35]. Therefore, Jang et al. previously investigated this concern in patients with IgA nephropathy [43]. They reported that the intrarenal compartment, and not the systemic pool, was the main source of urinary AGT even in patients with overt proteinuria. In our study, PSM was performed mostly in patients with microalbuminuria because the levels of UPCR were significantly lower in patients with PKD than in those with other etiologies of CKD. As the urinary AGT/Cr ratio can be increased in patients with overt proteinuria, further studies with a wide range of proteinuria are warranted to evaluate the RAAS activity in various etiologies of CKD with several components of the RAAS. Third, nutritional indices, including potassium intake, were not considered in our analyses. Low potassium intake is usually recommended for patients with CKD to avoid hyperkalemia in clinical practice, but it is also associated with high blood pressure and CKD progression [44, 45]. We did not include 24-h urinary potassium excretion in the analyses because it was measured only in 831 (46.5%) patients. However, when we compared 24-h urinary potassium excretion after PSM, the levels were not different between the PKD and non-PKD groups (data not shown).

## Conclusions

In conclusion, patients with PKD had a significantly lower serum potassium level than those with other etiologies of CKD. In PSM analysis, the urinary AGT/Cr ratio and TTKG were higher in patients with PKD than in those without PKD. In addition, the urinary AGT/Cr ratio was a significant prognostic marker for all-cause mortality or decline in renal function in patients with PKD. Therefore, further use of dual RAAS inhibition may be beneficial to reduce high intrarenal RAAS activity with a low risk of hyperkalemia in patients with PKD.

## Abbreviations

ACE: Angiotensin converting enzyme
ACEi: Angiotensin-converting enzyme inhibitor
AGT: Angiotensinogen
ARB: Angiotensin-II receptor blocker
BMI: Body mass index
CCI: Charlson comorbidity index
CI: Confidence interval
CKD: Chronic kidney disease
CO2: Carbon dioxide
Cr: Creatinine
DM: Diabetes mellitus
DN: Diabetic nephropathy
eGFR: Estimated glomerular filtration rate
GN: Glomerulonephritis
HALT-PKD: Halt Progression of Polycystic Kidney Disease
HR: Hazard ratio
HTN: Hypertensive nephrosclerosis
IDMS: Isotope dilution mass spectrometry
KNOW-CKD: KoreaN cohort study for Outcome in patients With CKD
PKD: Polycystic kidney disease
RAAS: Renin-angiotensin-aldosterone system
SBP: Systolic blood pressure
TTKG: Transtubular potassium gradient
UPCR: Urine protein-to-creatinine ratio
